# The Wiener index, degree distance index and Gutman index of composite hypergraphs and sunflower hypergraphs

**DOI:** 10.1016/j.heliyon.2022.e12382

**Published:** 2022-12-12

**Authors:** Sakina Ashraf, Muhammad Imran, Syed Ahtsham Ul Haq Bokhary, Shehnaz Akhter

**Affiliations:** a Centre for Advanced Studies in Pure and Applied Mathematics, Bahauddin Zakariya University, Multan, Pakistan; b Department of Mathematical Sciences, College of Sciences, United Arab Emirates University, Al Ain, United Arab Emirates; c School of Natural Sciences, National University of Sciences and Technology, H-12, Islamabad, Pakistan

**Keywords:** Wiener index, Degree distance index, Gutman index, Hypergraph, Sunflower hypergraph, Linear uniform hyper-paths

## Abstract

Topological invariants are numerical parameters of graphs or hypergraphs that indicate its topology and are known as graph or hypergraph invariants. In this paper, topological indices of hypergraphs such as Wiener index, degree distance index and Gutman index are considered. A *g*-composite hypergraphs is a hypergraphs that is obtained by the union of *g* hypergraphs with every hypergraph has exactly one vertex in common. In this article, results of above said indices for *g*-composite hypergraphs, where g≥2, are calculated. Further these results are used to find the Wiener index, degree distance index and Gutman index of sunflower hypergraphs and linear uniform hyper-paths.

## 1 Introduction

Chemists are accustomed to viewing molecules as geometric objects with a specific spatial arrangement of atoms. The positions of the atoms in the molecule are different, and a lot of types of intra-molecular motions are familiar to happen. On the other hand, despite all of these geometric changes, the molecule retains its identity. As a result, few things in the molecule must be unchanged when we made some changes in its geometry. Whenever the atomic motions are ignored, the geometry of a molecule is effected by its surroundings (like pressure, solvent choice, etc.). It has long been recognized that several characteristics of geometric shapes endure unchanged however continuously its points deformed. The theory was dubbed as “topology,” and it went on to become one of modern mathematics' most illustrious disciplines.

Although the topology of a molecule, as presented as the molecular graph, is an essentially non-numerical mathematical object, a lot of measurable characteristics of molecules are generally indicated numerically. To connect molecular topology with a real molecular characteristic, the statistics hold in the molecular graph must be first converted into a numerical characteristic. Topological indices are molecular invariants that present the structure or formation of molecules, helping to predict the activity and characteristics of molecules in complex experiments.

In computational chemistry and theoretical chemical research, graph representations of molecular structures are widely used [8], [14], [21]. Different topological indices of graphs, molecular graphs are widely studied in [5], [6], [7], [9], [10], [11], [12], [13], [16], [27]. Molecular structures are presented as graphs, with vertices and edges representing its atoms and its chemical bonds. In the research of ordinary graphs, the Molecular graphs has valuable importance [26]. Ordinary graphs, on the other hand, do not present chemical compounds with nonclassical structures. The lack of a appropriate presentation for molecules with delocalized polycentric bonds is a significant disadvantage of structure theory. Organometallic compounds are structures that contain one or more metal carbon bond [24]. Disconnected molecular graphs are sometimes used to represent ‘sandwich’ and oleon structures. Molecular subgraphs presenting individual structural particles in this representation, it does not appear to be illustrative and does not allow for the analysis of a structure as a whole. The connected molecular graphs are more illustrative, but not without drawbacks, in that all vertices corresponding to carbon atoms are joined the metal atom. In this case, the degree of the metal vertex is the cardinality of joined vertices and is not essential same to valency of metal atom. Furthermore, the difference between simple covalent and polycentric bonds is hidden in both representations. All of the above-mentioned structure presentation flaws are remove when hypergraphs are employed to present structures with delocalized polycentric bonds [29].

Hypergraph theory is playing an increasingly important role in chemistry, especially for depiction of non classical molecular structure with polycentric de-localized bonds [22], [28], [29]. The study in [28] shows that the hypergraph model is much accurate than graph model and hence tendency to give better description of the behavior of its invariants.

This paper will only consider unlabeled hypergraphs. In [28], a lot of methods of labeled hypergraph presentation of molecular structures with differing levels of detail and label types were considered. Let us now revisit some basic concepts of hypergraph theory.

## 2 Preliminaries

A hypergraph H compose of a non-empty vertex set V(H) and a given edge set X(H), where each edge e∈X(H) (refer to as a hyperedge) is a non-empty subset of V(H). Also, |V(H)| is the order of a given hypergraph H. For an integer m≥2, if the quantity of vertices incident to a hyperedge is exactly *m*, then hypergraph is known as a *m*-uniform. If s,u∈V(H) are distinct vertices and $e_{i}\in X(H)$ such that $s,u\in e_{i}$, then *s* and *u* are notable as linked in H through an edge $e_{i}$. In the same way, if $e_{i}$ and $e_{j}$ are distinct edges and s∈V(H), such that $s\in e_{i}\cap e_{j}$ then $e_{i}$ and $e_{j}$ are recognized as linked in H through vertex *s*. The order pair $\left(s,e_{i}\right)$, such that s∈V(H), $e_{i}\in X(H)$ and $s\in e_{i}$, is presented flag of H, and the set of flag is interpreted as $F_{H}$. The degree of s∈V(H) represented by $deg_{s}(H)$, is the cardinality of hyperedges that have *s*. The distance D(s,u) among two vertices *s* and *u* is the number of hyperedges in the shortest path that connects them. The vertices that have distance one from each other are known as the coincident (or adjacent) vertices. If H be a hypergraph and $F_{H}$ is a flag set then *Handshaking lemma* can be extended as:

$$\sum_{x\in V(H)}deg_{x}(H)=|F_{H}|.$$

A hypergraph $H^{\prime }$ is known as a hypersubgraph of H if $V^{\prime }(H^{\prime })\subset V(H)$ and $X^{\prime }(H^{\prime })\subset X(H)$. A hypergraph is connected if each pair of its vertices are joined by a path in H. In context of vertex connection, there is an equivalence relation on V(H) of hypergraph H. Let $V_{1}\subset V(H)$ be an equivalence class w.r.t. vertex connection then hypersubgraph induced by $V_{1}$ is recognized as a connected component of H. The quantity of given connected components of H is given by ω(H). A cut vertex is a vertex y∈V(H) such that ω(H∖y)>ω(H). Moreover, a vertex s∈V(H) is a cut vertex for H if H converted into two or more than two nonempty connected hypersubgraphs with just vertex *s* in common i.e. $H=H_{1}\cdot H_{2}$, where $H_{1}$ and $H_{2}$ are two nonempty connected hypersubgraphs of H with $V(H_{1})⋂V(H_{2})=\{s\}$. Let $H^{\prime }=H_{1}\cdot H_{2}\cdots H_{n}$, be a hypergraph constructed by n-hypersubgraphs $H_{1}\cdot H_{2}\cdots H_{n}$, such that $V(H_{1}\cdot H_{2}\cdots H_{n})=V(H_{1})\cup V(H_{2})\cup \cdots \cup V(H_{n})$ having one vertex common and edge set is $X(H_{1}\cdots H_{2}\cdots H_{n})={⋃}_{i=1}^{n}H_{i}$ is called n-composite hypergraph.

Wiener index is considered as the first generation of topological indices. It was introduced first time by Harry Wiener in 1947 [35]. Wiener, who was a chemist, introduced the graph's term path number in the form of sum of all distances among two atoms of carbon in molecules. Since then, there is a wide history and work on Wiener index of certain graphs [14], [25], [32], [33].

The Wiener index Win(H) of a simple connected hypergraph H is interpreted as:

(1) $$Win(H)=\sum_{\left\{s,y\}\subseteq V(H\right)}D(s,y)=\frac{1}{2}\sum_{s\in V(H)}\sum_{y\in V(H)}D(s,y).$$

The Wiener index of a given vertex *s* in H follows:

(2) $$win(s,H)=\sum_{y\in V(H)}D(s,y).$$

Thus,

$$Win(H)=\frac{1}{2}\sum_{s\in V(H)}win(s,H).$$

The degree distance index for a given graph was discussed by Dobrynin and Kochetova [15]. Also its discussed with different name by Gutman [21]. The degree distance of a hypergraph H [20] is written:

(3) $$DD(H)=\sum_{\{x,y\}\subseteq V(H)}(deg_{x}(H)+deg_{y}(H))D(x,y).$$

The degree distance of x∈V(H) in H is represented as:

(4) $$dd(x,H)=\sum_{y\in V(H)}D(x,y)deg_{y}(H).$$

The Gutman index for a given graph was presented in [20] and also extensively studied, see [1], [4], [17], [30], [31]. The Gutman index of a simple hypergraph H is interpreted:

(5) $$Gut(H)=\sum_{\{s,y\}\subseteq V(H)}deg_{s}(H)deg_{y}(H)D(s,y)=\frac{1}{2}\sum_{s\in V(H)}\sum_{y\in V(H)}deg_{s}(H)deg_{y}(H)D(s,y).$$

Sun et al. [34] found the Wiener index of a family of *m*-uniform hypergraphs and established a bound of Wiener index of these hypergraphs. In [19], Guo et al. determined *m*-uniform unique hypertrees with maximal Wiener indices of order up to three, also the unique *m*-uniform hypertrees with minimal Wiener indices of order up to three, respectively. They also gave some useful relations of degree distance index and Gutman index of hypertrees in [20].

A hypergraph is said to be a simple hypergraph if it has no loop and no multiple edges. Through out this paper, we consider hypergraphs which are simple and connected.

Our main goal in this work is to determine the Wiener, degree distance and Gutman indices of n-composite hypergraphs. This will generalize the corresponding result for graphs [2], [23]. As an application of n-composite hypergraphs, the above mentioned topological indices for sunflower hypergraph, linear uniform hyper-path, and linear uniform hyper-cycle have been obtained. Further a sunflower hypergraph $S_{g}^{n}$ having *g*-vertices common (core of the hypergraph) is defined and by direct calculation formulae of above said indices has been calculated.

## 3 Main results

The definition of composed graph is given by Hagri et al. in [2], they also proved certain important results.

Lemma 3.1 [2] *Let* $H=H_{1}\cdot H_{2}$ *be a graph composed by* $H_{1}$ *and* $H_{2}$ *graphs, having* $V(H_{1}\cdot H_{2})=V(H_{1})\cup V(H_{2})$ *and* $V(H_{1})\cap V(H_{2})=\{s\}$ *, where s is a cut vertex in* H *. Let* $|V(H_{1})|=t_{1}$ *and* $|V(H_{2})|=t_{2}$ *. If* $x\in V(H_{1})$ *and* $z\in V(H_{2})$ *, then we have*

*(1).* D(x,z)=D(x,s)+D(s,z) *.*
*(2).* $win(s,H)=win(s,H_{1})+win(s,H_{2})$ *.*
*(3).* $Win(H)=Win(H_{1})+Win(H_{2})+(t_{1}-1)win(s,H_{2})+(t_{2}-1)win(s,H_{1})$ *.*
*Similarly, if* H *is composed by g subgraphs* $H_{1},\cdots ,H_{g}$ *having number of vertices* $t_{1},\cdots ,t_{g}$ *, respectively, represented by* $H=H_{1}\cdot H_{2}\cdots H_{g}$ *, such that* $V(H)=\overset{g}{\underset{i=1}{⋃}}V(H_{i})$ *, and* $V(H_{b})\cap V(H_{h})=\{s\}$ *, for all* b≠h *, then*
*(4).* $Win(H)=\sum_{b=1}^{g}Win(H_{b})+\sum_{b=1}^{g-1}\sum_{h=b+1}^{g}\left((b_{h}-1)win(s,H_{b})+(t_{b}-1)win(s,H_{h})\right)$ *.*

In the next two theorems, this result has been extended for hypergraphs.

Theorem 3.1 *Let*H*be a hypergraph composed of g hypersubgraphs*$H_{1},H_{2},\cdots ,H_{g}$*having number of vertices*$t_{1},t_{2},\cdots ,t_{g}$*, respectively, such that*$V(H)=\overset{g}{\underset{i=1}{⋃}}V(H_{i})$*,*$V(H_{b})\cap V(H_{h})=\{s\}$*for all*b≠h*. If*$x\in V(H_{b})\setminus \{s\}$*, then*

*(1).* $win(x,H)=win(x,H_{b})+\sum_{h=1,h\neq b}^{g}(t_{h}-1)D(x,s)+\sum_{h=1,h\neq b}^{g}win(s,H_{h})$*.*
*(2).* $dd(x,H)=dd(x,H_{b})+\sum_{h=1,h\neq b}^{g}dd(s,H_{h})+D(x,s)\sum_{h=1,h\neq b}^{g}F_{H_{h}}$*.*

Proof (1). Let $x\in V(H_{b})\setminus \{s\}$. Then from Equation (2), we acquire

$$\begin{array}{cc} win(x,H) & =\sum_{z\in V(H)}D(x,z)\\ & =\left(\sum_{z\in V(H_{1})}D(x,z)+\ldots +\sum_{z\in V(H_{b})}D(x,z)+\ldots +\sum_{z\in V(H_{g})}D(x,z)\right)-(g-1)D(x,s)\\ & =win(x,H_{b})+\left(\sum_{h=1,h\neq b}^{g}\sum_{z\in V(H_{h})}(D(x,s)+D(s,z))\right)-(g-1)D(x,s)\\ & =win(x,H_{b})+\sum_{h=1,h\neq b}^{g}D(x,s)\sum_{z\in V(H_{h})}(1)+\sum_{h=1,h\neq b}^{g}\sum_{z\in V(H_{h})}D(s,z)-(g-1)D(x,s)\\ & =win(x,H_{b})+\sum_{h=1,h\neq b}^{g}t_{h}\cdot D(x,s)-(g-1)D(x,s)+\sum_{h=1,h\neq b}^{g}win(s,H_{h})\\ & =win(x,H_{b})+\left(\sum_{h=1,h\neq b}^{g}t_{h}-g+1\right)D(x,s)+\sum_{h=1,h\neq b}^{g}win(s,H_{h}) \end{array}$$

Hence the result

$$\begin{array}{cc} win(x,H) & =win(x,H_{b})+\sum_{h=1,h\neq b}^{g}(t_{h}-1)D(x,s)+\sum_{h=1,h\neq b}^{g}win(s,H_{h}). \end{array}$$

(2). Let $x\in V(H_{b})\setminus \{s\}$. Then from Equation (4), we get

$$\begin{array}{cc} dd(x,H) & =\sum_{z\in V(H)\setminus \{s\}}D(z,x)deg_{z}(H)+D(x,s)deg_{s}(H)\\ & =\sum_{z\in V(H_{1})\setminus \{s\}}D(z,x)deg_{z}(H_{1})+\sum_{z\in V(H_{b})\setminus \{s\}}D(z,x)deg_{z}(H_{b})+\cdots \\ & +\sum_{z\in V(H_{g})\setminus \{s\}}D(z,x)deg_{z}(H_{g})+D(s,x)deg_{s}(H)\\ & =\sum_{h=1,h\neq b}^{g}\sum_{z\in V(H_{h})\setminus \{s\}}D(z,x)deg_{z}(H_{h})+\sum_{z\in V(H_{b})\setminus \{s\}}D(z,x)deg_{z}(H_{b})+D(x,s)\sum_{h=1}^{g}deg_{s}(H_{h}) \end{array}$$

Since D(z,x)=D(z,s)+D(s,x), therefore

$$\begin{array}{cc} dd(x,H) & =\sum_{h=1,h\neq b}^{g}\sum_{z\in V(H_{h})\setminus \{s\}}\left(D(z,s)+D(s,x)\right)deg_{z}(H_{h})+\sum_{z\in V(H_{b})\setminus \{s\}}(D(z,x)deg_{z}(H_{b})\\ & +D(s,x)deg_{s}(H_{h})-D(s,x)deg_{s}(H_{h}))+D(s,x)\sum_{h=1}^{g}deg_{s}(H_{g})\\ & =dd(x,H_{b})+D(s,x)\left(\sum_{h=1,h\neq b}^{g}\sum_{z\in V(H_{h})\setminus \{s\}}deg_{z}(H_{h})\right)+\sum_{h=1,h\neq b}^{g}dd(s,H_{b})\\ & +D(x,s)\sum_{h=1,h\neq b}^{g}deg_{s}(H_{h}) \end{array}$$

Since $\sum_{z\in V(H_{h})\setminus \{s\}}deg_{z}(H_{h})=F_{H_{h}}-deg_{s}(H_{h})$, $\deg_{s}(H)=\sum_{h=1}^{g}deg_{s}(H_{h})$. Therefore

$$dd(x,H)=dd(x,H_{b})+\sum_{h=1,h\neq b}^{g}dd(s,H_{h})+D(x,s)\sum_{h=1,h\neq b}^{g}F_{H_{h}}.$$

After some simplification, we acquire the result. □

Theorem 3.2 *Let*H*be a hypergraph composed of g hypersubgraphs*$H_{1},H_{2},\cdots ,H_{g}$*having number of vertices*$t_{1},t_{2},\cdots ,t_{g}$*, respectively, such that*$V(H)=\overset{g}{\underset{i=1}{⋃}}V(H_{i})$*and*$V(H_{b})\cap V(H_{h})=\{s\}$*for all*b≠h*. Then*

*(1).* $Win(H)=\sum_{b=1}^{g}Win(H_{b})+\sum_{b=1}^{g-1}\sum_{h=b+1}^{g}\left((t_{b}-1)win(s,H_{h})+(t_{h}-1)win(s,H_{b})\right)$*.*
*(2).* $DD(H)=\sum_{b=1}^{k}DD(H_{b})+\sum_{b=1}^{g-1}\sum_{h=b+1}^{g}\left((t_{b}-1)dd(s,H_{h})+(t_{h}-1)dd(s,H_{b})\right)+\sum_{b=1}^{g-1}\sum_{h=b+1}^{g}(win(s,H_{b})F_{H_{h}}+win(s,H_{h})F_{H_{b}})$*.*
*(3).* $Gut(H)=\sum_{b=1}^{g}Gut(H_{b})+\sum_{b=1}^{g-1}\sum_{h=b+1}^{g}\left(F_{H_{h}}dd(s,H_{b})+F_{H_{b}}dd(s,H_{h})\right)$*.*

Proof (1). From Equation (1), we acquire

$$\begin{array}{cc} Win(H) & =\sum_{\left\{x,z\}\subset V(H\right)}D(x,z)=\frac{1}{2}\sum_{x\in V(H)}\sum_{z\in V(H)}D(x,z)\\ & =\left(\frac{1}{2}\sum_{x\in V(H_{1})}\sum_{z\in V(H_{1})}D(x,z)+\cdots +\frac{1}{2}\sum_{x\in V(H_{g})}\sum_{z\in V(H_{g})}D(x,z)\right)\\ & +\left(\sum_{x\in V(H_{g-1})\setminus \{s\}}\sum_{z\in V(H_{g})\setminus \{s\}}D(x,z)\right)+(\sum_{x\in V(H_{g-2})\setminus \{s\}}\sum_{z\in V(H_{g-1})}D(x,z)\\ & +\sum_{x\in V(H_{g-2})\setminus \{s\}}\sum_{z\in V(H_{g})\setminus \{s\}}D(x,z))+\cdots +(\sum_{x\in V(H_{b})\setminus \{s\}}\sum_{z\in V(H_{b+1})\setminus \{s\}}D(x,z) \end{array}$$

$$\begin{array}{cc} & +\cdots +\sum_{x\in V(H_{b})\setminus \{s\}}\sum_{z\in V(H_{n})\setminus \{s\}}D(x,z))+\cdots +(\sum_{x\in V(H_{1})\setminus \{s\}}\sum_{z\in V(H_{2})\setminus \{s\}}D(x,z)\\ & +\cdots +\sum_{x\in V(H_{1})\setminus \{s\}}\sum_{z\in V(H_{g})\setminus \{s\}}D(x,z))\\ & =\sum_{b=1}^{g}Win(H_{b})+\sum_{b=1}^{g-1}\sum_{h=b+1}^{g}\left(\sum_{x\in V(H_{b})\setminus \{s\}}\sum_{z\in V(H_{h})\setminus \{s\}}D(x,z)\right). \end{array}$$

Since D(x,z)=D(x,s)+D(s,z), therefore

$$\begin{array}{cc} \sum_{x\in V(H_{b})\setminus \{s\}}\sum_{z\in V(H_{h})\setminus \{s\}}D(x,z) & =\sum_{x\in V(H_{b})\setminus \{s\}}\sum_{z\in V(H_{h})\setminus \{s\}}\left(D(x,s)+D(s,z)\right)\\ & =\sum_{x\in V(H_{b})\setminus \{s\}}\sum_{z\in V(H_{h})\setminus \{s\}}D(x,s)+\sum_{x\in V(H_{b})\setminus \{s\}}\sum_{z\in V(H_{h})\setminus \{s\}}D(s,z)\\ & =(t_{h}-1)\sum_{x\in V(H_{b})\setminus \{s\}}D(x,s)+(t_{b}-1)\sum_{z\in V(H_{h})\setminus \{s\}}D(s,z)\\ & =(t_{h}-1)win(s,H_{b})+(t_{b}-1)win(s,H_{h}). \end{array}$$

This implies that

$$\begin{array}{cc} Win(H) & =\sum_{b=1}^{k}Win(H_{b})+\sum_{b=1}^{g-1}\sum_{h=b+1}^{g}\left((t_{h}-1)win(s,H_{b})+(t_{b}-1)win(s,H_{h})\right). \end{array}$$

(2). By using Equation (2) in the definition of degree distance given in Equation (3), we have

$$\begin{array}{cc} DD(H) & =\frac{1}{2}\sum_{x\in V(H)}\sum_{z\in V(H)}(deg_{x}(H)+deg_{z}(H))D(x,z)=\sum_{x\in V(H)}\sum_{z\in V(H)}D(x,z)deg_{x}(H)\\ & =\sum_{x\in V(H_{1})\cup \ldots \cup V(H_{g})}win(x,H)deg_{x}(H)\\ & =(\sum_{x\in V(H_{1})\setminus \{s\}}win(x,H)deg_{x}(H_{1})+\sum_{x\in V(H_{2})\setminus \{s\}}win(x,H)deg_{x}(H_{2})+\cdots \\ & +\sum_{x\in V(H_{g})\setminus \{s\}}win(x,H)deg_{x}(H_{g}))+win(s,H)deg_{s}(H)\\ & =\sum_{b=1}^{g}\sum_{x\in V(H_{b})\setminus \{s\}}win(x,H)deg_{x}(H_{b})+win(s,H)deg_{s}(H). \end{array}$$

Note that $win(s,H_{1}\cdot H_{2}\cdots H_{g})=\sum_{b=1}^{g}win(s,H_{b})$ and using Theorem 3.1, we get

$$\begin{array}{cc} DD(H) & =\sum_{b=1}^{g}\sum_{x\in V(H_{b})\setminus \{s\}}\left\{win(x,H_{b})+\sum_{h=1,h\neq b}^{g}(t_{h}-1)D(x,s)+\sum_{h=1,h\neq b}^{g}win(s,H_{h})\right\}deg_{x}(H_{b})\\ & +\sum_{b=1}^{g}w(s,H_{b})deg_{s}(H) \end{array}$$

$$\begin{array}{cc} & =\sum_{b=1}^{g}(\sum_{x\in V(H_{b})\setminus \{s\}}win(x,H_{b})\cdot deg_{x}(H_{b})+\sum_{x\in V(H_{b})\setminus \{s\}}D(x,s)deg_{x}(H_{b})\sum_{h=1,h\neq b}^{g}(t_{h}-1)\\ & +\sum_{x\in V(H_{b})\setminus \{s\}}deg_{x}(H_{b})\sum_{h=1,h\neq b}^{g}win(s,H_{h}))+\sum_{b=1}^{g}win(s,H_{b})deg_{s}(H) \end{array}$$

Since $\sum_{x\in V(H_{b})\setminus \{s\}}deg_{x}(H_{b})=F_{H_{b}}-deg_{s}(H_{b})$ and $\sum_{x\in V(H_{b})}D(x,s)deg_{x}(H)=dd(s,H_{b})$. Therefore

$$\begin{array}{cc} DD(H) & =\sum_{b=1}^{g}\left(\sum_{x\in V(H_{b})\setminus \{s\}}win(x,H_{b})deg_{x}(H_{b})+win(s,H_{b})deg_{s}(H_{b})-win(s,H_{b})deg_{s}(H_{b})\right)\\ & +\sum_{b=1}^{g}dd(s,H_{b})\sum_{h=1,h\neq b}^{g}(t_{h}-1)+\sum_{b=1}^{g}(F_{H_{b}}-deg_{s}(H_{b}))\sum_{h=1,h\neq b}^{g}win(s,H_{h})+\sum_{b=1}^{g}win(s,H_{b})deg_{s}(H)\\ & =\sum_{b=1}^{g}DD(H_{b})+\sum_{b=1}^{g-1}\sum_{h=b+1}^{g}\left((t_{b}-1)dd(s,H_{h})+(t_{h}-1)dd(s,H_{b})\right)\\ & +\sum_{b=1}^{g}(F_{H_{b}}-deg_{s}(H_{b}))\sum_{h=1,h\neq b}^{g}win(s,H_{h})+\sum_{b=1}^{g}win(s,H_{b})\sum_{h=1,h\neq b}^{g}deg_{s}(H_{h})\\ & =\sum_{b=1}^{g}DD(H_{b})+\sum_{b=1}^{g-1}\sum_{h=b+1}^{g}\left((t_{b}-1)dd(s,H_{h})+(t_{h}-1)dd(s,H_{b})\right)\\ & +\sum_{b=1}^{g-1}\sum_{h=b+1}^{g}\{win(s,H_{b})F_{H_{h}})+win(s,H_{h})F_{H_{b}}\}. \end{array}$$

After simplification, we acquire the desire result.

(3). Using definition of degree distance of a vertex in (5), we can get Gutman index as

$$\begin{array}{cc} Gut(H) & =\sum_{\left\{x,z\}\subset V(H\right)}deg_{x}(H)deg_{z}(H)D(x,z)=\frac{1}{2}\sum_{x\in V(H)}\sum_{z\in V(H)}deg_{x}(H)deg_{z}(H)D(x,z)\\ & =\frac{1}{2}\sum_{x\in V(H)}dd(x,H)deg_{x}(H)\\ 2.Gut(H) & =\sum_{x\in V(H_{1})\setminus \{s\}}dd(x,H)deg_{x}(H_{1})+\sum_{x\in V(H_{2})\setminus \{s\}}dd(x,H)deg_{x}(H_{2})+\cdots \\ & +\sum_{x\in V(H_{g})\setminus \{s\}}dd(x,H)deg_{x}(H_{g})+dd(s,H)deg_{s}(H)\\ & =\sum_{b=1}^{g}\sum_{x\in V(H_{b})\setminus \{s\}}dd(x,H)deg_{x}(H_{b})+dd(s,H)deg_{s}(H). \end{array}$$

By using Theorem 3.1, we acquire

$$\begin{array}{cc} 2Gut(H) & =\sum_{b=1}^{g}\sum_{x\in V(H_{b})\setminus \{s\}}\left(dd(x,H_{b})+\sum_{h=1,h\neq b}^{g}dd(s,H_{h})+D(x,s)\sum_{h=1,h\neq b}^{g}F_{H_{h}}\right)\deg_{x}(H_{b})\\ & +dd(s,H)deg_{s}(H)\\ & =\sum_{b=1}^{g}\sum_{x\in V(H_{b})\setminus \{s\}}dd(x,H_{b})deg_{x}(H_{b})+\sum_{b=1}^{g}\sum_{x\in V(H_{b})\setminus \{s\}}deg_{x}(H_{b})\sum_{h=1,h\neq b}^{g}dd(s,H_{h})\\ & +\sum_{b=1}^{g}\sum_{x\in V(H_{b})\setminus \{s\}}D(x,s)deg_{x}(H_{b})\sum_{h=1,h\neq b}^{g}F_{H_{h}}+dd(s,H)deg_{s}(H)\\ & =\sum_{b=1}^{g}\sum_{x\in V(H_{b})}dd(x,H_{b})deg_{x}(H_{b})-\sum_{b=1}^{g}dd(s,H_{b})deg_{s}(H_{b})+\sum_{b=1}^{g}\sum_{x\in V(H_{b})\setminus \{s\}}deg_{x}(H_{b})\\ & \sum_{h=1,h\neq b}^{g}dd(s,H_{h})+\sum_{b=1}^{g}\sum_{x\in V(H_{b})\setminus \{s\}}D(x,s)deg_{x}(H_{b})\sum_{h=1,h\neq b}^{g}F_{H_{b}}+dd(s,H)deg_{s}(H), \end{array}$$

now using $\sum_{x\in V(H_{b})\setminus \{s\}}deg_{x}(H_{b})=F_{H_{b}}-deg_{s}(H_{b})$ and also by Equation (4), we acquire

$$\begin{array}{cc} 2Gut(H) & =2\sum_{b=1}^{g}Gut(H_{b})+\sum_{b=1}^{g}(F_{H_{b}}-deg_{s}(H_{b}))\sum_{h=1,h\neq b}^{g}dd(s,H_{h})\\ & +\sum_{b=1}^{g}dd(s,H_{b})\sum_{h=1,h\neq b}^{g}F_{H_{h}}+\sum_{b=1}^{g}dd(s,H_{b})\sum_{h=1,h\neq b}^{g}deg_{s}(H_{h})\\ & =2\sum_{b=1}^{g}Gut(H_{b})+2\sum_{b=1}^{g-1}\sum_{h=b+1}^{g}\left(F_{H_{h}}dd(s,H_{b})+dd(s,H_{h})F_{H_{b}}\right)\\ Gut(H) & =\sum_{b=1}^{g}Gut(H_{b})+\sum_{b=1}^{g-1}\sum_{h=b+1}^{g}\left(F_{H_{h}}dd(s,H_{b})+dd(s,H_{h})F_{H_{b}}\right) \end{array}$$

This finishes the result. □

## 4 Applications

In present section, we implement Theorem 3.2 to find the Wiener, degree distance and Gutman indices of sunflower hypergraphs and linear uniform hyper-paths.

### 4.1 Sunflower hypergraph

**Figure 1:**
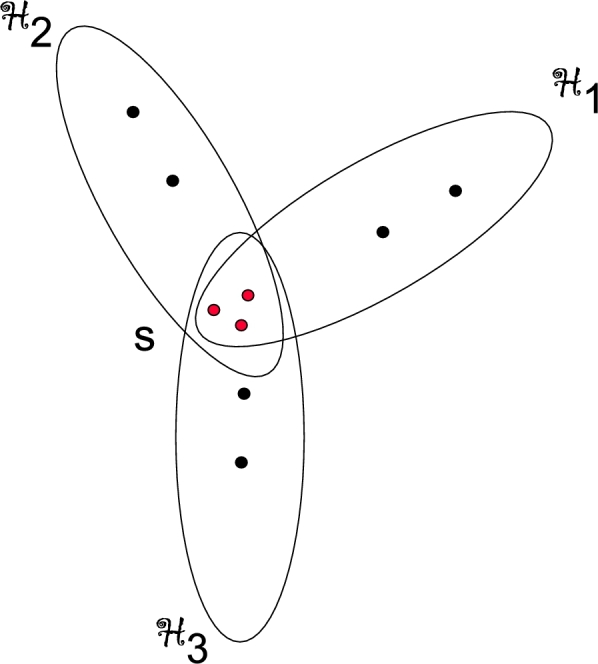
A 5-uniform sunflower $S_{3}^{3}$.

A sunflower hypergraph $S_{b}^{n}$ with n petals $H_{i}$ (1≤i≤n) and a core *S* with |V(S)|=b is a collection of sets $H_{1},H_{2},\cdots ,H_{n}$ such that $H_{b}\cap H_{h}=S$ for all b≠h [3]. The components of core *S* are presented as seeds. A Venn diagram of given sets be presented as a sunflower. Any family of pairwise disjoint sets is a sunflower (having an empty core) and a hyperstar having is a core of size 1 is known as a sunflower. Fig. 1 depicted a 5-uniform sunflower hypergraph having a core of size 3 and $H_{1},H_{2}$ and $H_{3}$ as petals.

In the next result, the Wiener, degree distance and Gutman indices of $S_{1}^{n}$ are determined by the help of Theorem 3.2.

Lemma 4.1 *The Wiener, degree distance and Gutman indices of*$S_{1}^{n}$*are*

*(1).* $Win(S_{1}^{n})=\frac{1}{2}\sum_{b=1}^{n}t_{b}(t_{b}-1)+2\sum_{b=1}^{n-1}\sum_{h=b+1}^{n}(t_{b}-1)(t_{h}-1)$*,*
*(2).* $DD(S_{1}^{n})=\sum_{b=1}^{n}(t_{b}-1)(t_{b}+n-1)+4\sum_{b=1}^{n-1}\sum_{h=b+1}^{n}\left((t_{b}-1)(t_{h}-1)\right)$*,*
*(3).* $Gut(S_{1}^{n})=\frac{1}{2}\sum_{b=1}^{n}(t_{b}-1)(t_{b}+2(n-1))+2\sum_{b=1}^{n-1}\sum_{h=b+1}^{n}(t_{b}-1)(t_{h}-1)$*.*

Proof If each $H_{b}$ in Theorem 3.2 is an hyper edge such that $|V(H_{b})|=t_{b}$ (1≤b≤n), then the resulting hypergraph H is isomorphic to $S_{1}^{n}$. Thus, we get

(6) $$Win(S_{1}^{n})=Win(H)=\sum_{b=1}^{n}Win(H_{b})+\sum_{b=1}^{n-1}\sum_{h=b+1}^{g}[(t_{b}-1)win(s,H_{h})+(t_{h}-1)win(s,H_{b})].$$

(7) $$\begin{array}{cc} DD(H)= & \sum_{b=1}^{n}DD(H_{b})+\sum_{b=1}^{n-1}\sum_{h=b+1}^{n}\left\{(t_{b}-1)dd(s,H_{h})+(t_{h}-1)dd(s,H_{b})\right\}\\ & +\sum_{b=1}^{n-1}\sum_{h=b+1}^{n}\{win(s,H_{b})(F_{H_{h}})+win(s,H_{h})(F_{H_{b}})\}. \end{array}$$

(8) $$Gut(H)=\sum_{b=1}^{n}Gut(H_{b})+\sum_{b=1}^{n-1}\sum_{h=b+1}^{n}\left(F_{H_{h}}dd(s,H_{b})+F_{H_{b}}dd(s,H_{h})\right).$$

Further, in this case we have $Win(H_{b})=\left(\begin{array}{c} t_{b}\\ 2 \end{array}\right)$ and for all 1≤b≤n, $dd(x,H_{b})=win(x,H_{b})=(t_{b}-1)$ and $F_{H_{b}}=t_{b}$. By replacing these in Equation (6), we get

$$\begin{array}{cc} Win(S_{1}^{n}) & =\sum_{b=1}^{n}\left(\begin{array}{c} t_{b}\\ 2 \end{array}\right)+\sum_{b=1}^{n-1}\sum_{h=b+1}^{n}((t_{b}-1)(t_{h}-1)+(t_{h}-1)(t_{b}-1))\\ & =\frac{1}{2}\sum_{b=1}^{n}t_{b}(t_{b}-1)+2\sum_{b=1}^{n-1}\sum_{h=b+1}^{n}(t_{b}-1)(t_{h}-1). \end{array}$$

From the construction of $S_{1}^{n}$ hypergraph, we have $DD(H_{b})=2\left(\begin{array}{c} t_{b}\\ 2 \end{array}\right)$ substituting in Equation (7) and get

$$\begin{array}{cc} DD(S_{1}^{n}) & =\sum_{b=1}^{n}DD(H_{b})+4\sum_{b=1}^{n-1}\sum_{h=b+1}^{n}((t_{b}-1)(t_{h}-1))+(n-1)\sum_{b=1}^{n}(t_{b}-1)\\ & =\sum_{b=1}^{n}(t_{b}-1)(t_{b}+n-1)+4\sum_{b=1}^{n-1}\sum_{h=b+1}^{n}\left((t_{b}-1)(t_{h}-1)\right). \end{array}$$

Similarly, from Equation (8), we get

$$\begin{array}{cc} Gut(S_{1}^{n}) & =\sum_{b=1}^{n}Gut(H_{b})+2\sum_{b=1}^{n-1}\sum_{h=b+1}^{n}((t_{b}-1)(t_{h}-1))+(n-1)\sum_{b=1}^{n}(t_{b}-1), \end{array}$$

where

$$\begin{array}{cc} \sum_{b=1}^{n}Gut(H_{b}) & =\sum_{b=1}^{n}\left(\begin{array}{c} t_{b}-1\\ 2 \end{array}\right). \end{array}$$

Thus, we get

$$\begin{array}{cc} Gut(S_{1}^{n}) & =\frac{1}{2}\sum_{b=1}^{n}(t_{b}-1)(t_{b}+2(n-1))+2\sum_{b=1}^{n-1}\sum_{h=b+1}^{n}(t_{b}-1)(t_{h}-1). \end{array}$$

This completes the proof. □

**Particular case:** If in $S_{1}^{n}$, hyper edges are of same order *m*, i.e., $(t_{b}=m$ for b=1,2,3,⋯,n), then

$$\left\{ \begin{array}{cc} win(x,H_{b})=win(x,H_{h})=win(x,H_{m})\\ H_{b}=H_{h}=H_{m} & for\;b,h\in \{1,2,3,\cdots ,n\}\\ N=n(m-1)+1 \end{array}\right.$$

In this case, Lemma 4.1 reduces to following form.

Corollary 4.1

*(1).* $Win(S_{1}^{n})=\frac{1}{2}(N-1)(2N-m)$
*(2).* $DD(S_{1}^{n})=(N-1)(2N-m+n-1)$
*(3).* $Gut(S_{1}^{n})=\frac{1}{2}m(N-1)(2n-1)$

Let $S_{g}^{n}$ be a sunflower hypergraph composed of n edges $\left(H_{1},\ldots ,H_{n}\right)$, |V(S)|=g, where g≥2. Then sunflower hypergraph $S_{g}^{n}$ has *g* elements in the core and n is the quantity of edges.

In this case, the Wiener, degree distance and Gutman indices of $S_{g}^{n}$ are computed in upcoming result.

Theorem 4.1 *Let*$S_{g}^{n}$*be a sunflower hypergraph with*n*edges*$\left(H_{1},\ldots ,H_{n}\right)$*of cardinality*$t_{b}$*,*b=1,2,…n*, respectively. Then, for*g,n≥2

*(1).* $Win(S_{g}^{n})=\frac{1}{2}\sum_{b=1}^{n}t_{b}(t_{b}-g)+2\sum_{b=1}^{n}\sum_{h=b+1}^{n}(t_{b}-g)(t_{h}-g)-\frac{g(g-1)(n-1)}{2}$*,*
*(2).* $DD(S_{g}^{n})=\sum_{b=1}^{n}(t_{b}-g)(t_{b}+kn-1)+nk(g-1)+4\sum_{b=1}^{n-1}\sum_{h=b+1}^{n}(t_{b}-g)(t_{h}-g)$*,*
*(3).* $Gut(S_{g}^{n})=\frac{1}{2}\sum_{b=1}^{n}(t_{b}-g)(t_{b}+g(2n-1)-1)+n^{2}\frac{g(g-1)}{2}+2\sum_{b=1}^{n-1}\sum_{h=b+1}^{n}(t_{b}-g)(t_{h}-g)$*.*

**Figure 2:**
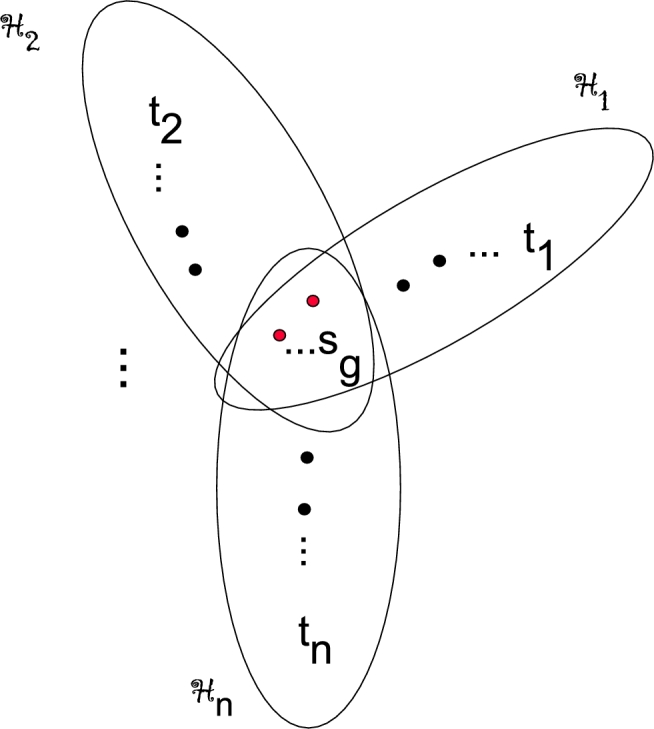
Sunflower hypergraph $S_{g}^{n}$.

Proof The order of sunflower hypergraph $S_{g}^{n}$ (see Fig. 2) is $N=\sum_{b=1}^{n}t_{b}-g(n-1)$ and order of each hyper edge is $t_{b}=|V(H_{b})|$, b=1,…n. Since, there are *g* vertices which are common in each $H_{b}$ (1≤b≤n) therefore $\overset{n}{\underset{b=1}{⋂}}H_{b}=S$. Let $l=t_{b}-g$ be number of vertices of $H_{b}$ which are not in *S*. From the construction of $S_{g}^{n}$, the pair of vertices can be partitioned on the basis of their distances from each other and there degrees, shown in the Table 1.

**Table 1:** Partitioning of pair of vertices w.r.t. their degree and distances from each other.

(*deg*_*x*_,*deg*_*z*_)	*D*(*x*,*z*)	Cardinality
(1,1)	1	$\sum_{b=1}^{n}\left(\begin{array}{c} t_{b}-g\\ 2 \end{array}\right)$
(1,1)	2	$\sum_{b=1}^{n-1}\sum_{h=b+1}^{n}(t_{b}-g)(t_{h}-g)$
(1,n)	1	$\sum_{b=1}^{n}g(t_{b}-g)$
(n,n)	1	$\left(\begin{array}{c} g\\ 2 \end{array}\right)$

(1). From Equation (1), we acquire

$$\begin{array}{cc} Win(S_{g}^{n}) & =\sum_{x,z\in e_{b}}D(x,z)+\sum_{x\in e_{b},z\in e_{h},h\neq b}D(x,z) \end{array}$$

Using information given in Table 1, we get

$$\begin{array}{c} Win(S_{g}^{n})=\sum_{b=1}^{n}\left(\begin{array}{c} t_{b}-g\\ 2 \end{array}\right)\cdot 1+\sum_{b=1}^{n-1}\sum_{h=b+1}^{n}(t_{b}-g)(t_{h}-g)\cdot 2+\sum_{b=1}^{n}g(t_{b}-g)\cdot 1+\left(\begin{array}{c} g\\ 2 \end{array}\right)\cdot 1 \end{array}$$

After simplification we get the required result.

$$\begin{array}{c} Win(S_{g}^{n})=\frac{1}{2}\sum_{b=1}^{n}t_{b}(t_{b}-g)+2\sum_{b=1}^{n}\sum_{h=b+1}^{n}(t_{b}-g)(t_{h}-g)-\frac{g(g-1)(n-1)}{2}. \end{array}$$

Similarly, (2) and (3) can be proved easily by using definition of degree distance (see [16]), and Table 1 and definition of Gutman index and Table 1, respectively. □

**Particular cases:** If $S_{g}^{n}$ is uniform hypergraph, i.e. each edge contain same number of vertices say *m* ($H_{b}=m$ for b=1,⋯,n), then Theorem 4.1 reduces into following form.

Corollary 4.2 *The Wiener, degree distance and Gutman indices for uniform sunflower*$S_{g}^{n}$*are*

*(1).* $Win(S_{g}^{n})=\frac{1}{2}\left((N-g)(N-m)+N(N-1)\right)$*,*
*(2).* $DD(S_{g}^{n})=(N-g)\{2N-m-1\}+gn(N-1)$*,*
*(3).* $Gut(S_{g}^{n})=\frac{1}{2}(N-g)\left(2(N-1)+2gn-m-1\right)$*.*

### 4.2 Linear uniform hyper path

**Figure 3:**
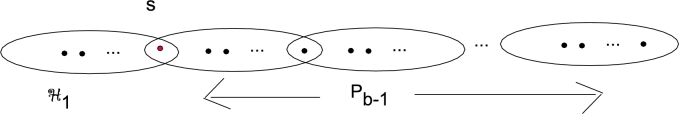
A linear uniform hyper-path $P_{b}^{n}$.

A linear uniform hyper-path $P_{b}^{n}$ (see Figure 3) is a collection of sets $H_{1},H_{2},\cdots ,H_{b}$ such that $|V(H_{b})\cap V(H_{b+1})|=1$ and $|V(H_{b})|=n$ [18]. In the next result, the Wiener, degree distance and Gutman indices of $P_{b}^{n}$ are determined by the help of Theorem 3.2.

Lemma 4.2 *The Wiener, degree distance and Gutman indices of*$P_{b}^{n}$*are*

*(1).* $Win(P_{b}^{n})=b\left(\begin{array}{c} n\\ 2 \end{array}\right)+\frac{b(b-1)(b+4)}{6}{\left(n-1\right)}^{2}$*,*
*(2).* $DD(P_{b}^{n})=(n-1)\{(n-2)\left(\begin{array}{c} b\\ 2 \end{array}\right)+n\frac{b(b+1)(2b+1)}{6}\}$*,*
*(3).* $Gut(P_{b}^{n})=\frac{bn}{2}\{\left((b+1)(b+2)-3\right)\frac{n}{3}-\left(2b-1\right)\}$*.*

## 5 Conclusion

In this article, we have provided the formulae for computing the Wiener index, degree-distance index and Gutman index of n-composite hypergraphs. Since, every graph is a 2-uniform hypergraph. Therefore, these results will also extend the corresponding previous results for graphs. As an application of n-composite hypergraphs, the above mentioned topological indices for sunflower hypergraph and linear uniform hyper-path, have been obtained.

The ordinary graphs do not represent chemical compounds with non-classical structures. The flaws of these structure representation can be eliminated when we used hypergraphs for the representation of structures with delocalized polycentric bonds. In future, it will be interesting model the hypergraphs from different chemical structures and then explore different topological properties. It is also worthy to study different physical properties of these hypergraphs and correlate them to different topological indices such as Wiener index, degree distance index and Gutman index. We are also hoping to extend these results to parallel composition and then to series parallel composition of hypergraphs.

## Declarations

### Author contribution statement

Sakina Ashraf: Conceived and designed the analysis; Wrote the paper.

Muhammad Imran: Conceived and designed the analysis; Contributed analysis tools or data.

Syed Ahtsham Ul Haq Bokhary: Analyzed and interpreted the data; Wrote the paper.

Shehnaz Akhter: Analyzed and interpreted the data; Contributed analysis tools or data.

### Funding statement

Dr. Muhammad Imran was supported by 10.13039/501100006013United Arab Emirates University [G00003461].

### Data availability statement

No data was used for the research described in the article.

### Declaration of interests statement

The authors declare no conflict of interest.

### Additional information

No additional information is available for this paper.
